# A very-hot food and beverage thermal exposure index and esophageal cancer risk in Malawi and Tanzania: findings from the ESCCAPE case–control studies

**DOI:** 10.1038/s41416-022-01890-8

**Published:** 2022-06-29

**Authors:** Gwinyai Masukume, Blandina T. Mmbaga, Charles P. Dzamalala, Yohannie B. Mlombe, Peter Finch, Gissela Nyakunga-Maro, Alex Mremi, Daniel R. S. Middleton, Clement T. Narh, Steady J. D. Chasimpha, Behnoush Abedi-Ardekani, Diana Menya, Joachim Schüz, Valerie McCormack

**Affiliations:** 1 Environment and Lifestyle Epidemiology Branch, International Agency for Research on Cancer, World Health Organization, Lyon, France; 2 Kilimanjaro Clinical Research Institute, Kilimanjaro Christian Medical Centre and Kilimanjaro Christian Medical University College, Moshi, Tanzania; 3 Malawi College of Medicine, Blantyre, Malawi; 4 School of Public Health, University of Health and Allied Sciences, Hohoe, Ghana; 5 Faculty of Epidemiology and Population Health, London School of Hygiene & Tropical Medicine, London, UK; 6 Genomic Epidemiology Branch, International Agency for Research on Cancer, World Health Organization, Lyon, France; 7 School of Public Health, Moi University, Eldoret, Kenya

**Keywords:** Risk factors, Cancer epidemiology

## Abstract

**Background:**

Consumption of very-hot beverages/food is a probable carcinogen. In East Africa, we investigated esophageal squamous cell carcinoma (ESCC) risk in relation to four thermal exposure metrics separately and in a combined score.

**Methods:**

From the ESCCAPE case–control studies in Blantyre, Malawi (2017-20) and Kilimanjaro, Tanzania (2015-19), we used logistic regression models adjusted for country, age, sex, alcohol and tobacco, to estimate odds ratios (ORs) and 95% confidence intervals (CIs) for self-reported thermal exposures whilst consuming tea, coffee and/or porridge.

**Results:**

The study included 849 cases and 906 controls. All metrics were positively associated with ESCC: temperature of drink/food (OR 1.92 (95% CI: 1.50, 2.46) for ‘very hot’ vs ‘hot’), waiting time before drinking/eating (1.76 (1.37, 2.26) for <2 vs 2–5 minutes), consumption speed (2.23 (1.78, 2.79) for ‘normal’ vs ‘slow’) and mouth burning (1.90 (1.19, 3.01) for ≥6 burns per month vs none). Amongst consumers, the composite score ranged from 1 to 12, and ESCC risk increased with higher scores, reaching an OR of 4.6 (2.1, 10.0) for scores of ≥9 vs 3.

**Conclusions:**

Thermal exposure metrics were strongly associated with ESCC risk. Avoidance of very-hot food/beverage consumption may contribute to the prevention of ESCC in East Africa.

## Introduction

According to global cancer burden 2020 estimates, esophageal cancer (EC) is the sixth most common cause of death (544,076 deaths) from cancer and the eighth-most common cancer (604,100 new cases) [1]. Those diagnosed with EC have an unfavourable prognosis, for instance a 20% 5-year survival in the United States of America [2]. There are two major histological subtypes of EC, these are esophageal adenocarcinoma and squamous cell carcinoma (ESCC) [3]. The predominant type globally is ESCC (~88%), which is also the dominant type in geographical hotspots such as parts of China, Iran and the eastern African corridor. This corridor stretches from Ethiopia [4] to South Africa [5] and includes several countries, for example, Kenya, Malawi, Tanzania, Zambia and Zimbabwe. In 2020 estimates, the age standardised incidence rates for EC were 20.3 and 15.2 per 100,000 person-years for males and females respectively in Malawi. The corresponding figures for Tanzania were 12.8 and 6.6.

An extensive review of putative aetiologic risk factors for ESCC in Africa suggests that many carcinogens and susceptibility factors that contribute to the Chinese and Iranian hotspots may also be present in Africa, with setting-specific exposure habits or sources [6]. Studies addressing these factors have been undertaken in the past decade, now forming a strong element of the African Esophageal Cancer Consortium [7]. To date, results point to the increased risks associated with tobacco, alcohol, poor oral health, household air pollution and the reduced intake of fruit and vegetables [8–14]. Another putative determinant for ESCC is thermal injury from the consumption of very-hot food and beverages [3].

In 2016, the consumption of very-hot beverages (>65 °C) was evaluated as Group 2A “probably carcinogenic to humans” by the International Agency for Research on Cancer (IARC) monographs working group [15]. Since this IARC monograph, two prospective cohort studies in Iran and China have also reported positive associations [16, 17]. In Iran, ESCC incidence rates were raised with a higher measured first sip temperature (relative risk (RR) 1.41 for >60 °C vs <60 °C) and were even stronger for self-reported temperature (RR 2.4 for ‘very hot’ vs ‘cold/lukewarm’) [16]. Evidence of positive links between thermal injury and ESCC have also been reported in Africa, in 2019 in a case–control study from Kenya [18]. In contrast in a Tanzanian case–control study [10] no association was found for either preferred beverage temperature or for burning the mouth in the past year (odds ratios ~1.1 for both). There are, however, two cross-sectional studies in Eastern Africa suggesting that hot beverage consumption temperatures are extremely high, with mean first sip tea temperatures of 70.6 °C in Tanzania [19] and 72.6 °C in neighbouring Kenya [20]. In the Tanzanian study, milky tea (50% high-fat milk and 50% water) was consumed 1.9 °C hotter than those who drank black tea because it had cooled at a slower rate [19]. Two further aspects of thermal injury are important to highlight. First, effect modification was found in the aforementioned Chinese prospective cohort, with a stronger thermal injury effect among those who also smoked tobacco or drank alcohol [17]. Second, thermal exposure metrics need to extend beyond the temperature of the first sip, to include sip volume and temperature throughout the drinking episode, as shown in early studies of their influences on the intra-esophageal temperature [21]. Notably, a larger sip at a lower temperature can lead to the same intra-esophageal liquid temperature as a smaller sip at a higher temperature.

In the current case–control study, which was conducted in Malawi and Tanzania, we investigated self-reported markers of thermal exposures during the ingestion of food/drink and its association with the risk of ESCC. The study benefitted from a more detailed exposure assessment than in previous studies, which we used to generate a thermal exposure index. Explicitly, we collected the data on a range of hot beverages/foods, i.e., porridge, tea, and coffee and whether and what type of milk was added, and several exposure correlates beyond self-perceived drinking temperature, including drinking frequency, speed and volume, waiting time prior to consumption and mouth/tongue burning frequency.

## Materials and methods

### Study setting

As part of the Esophageal Squamous Cell Carcinoma African Prevention Research (ESCCAPE) programme, hospital-based case–control studies of ESCC were conducted in Kenya, Malawi and Tanzania. The Kenya findings on ESCC and hot beverages were previously published, as already mentioned [18], and herein we analysed the data from Malawi and Tanzania. The Malawian study took place at the tertiary Queen Elizabeth Central Hospital, which has endoscopy services, in Blantyre the capital city of Malawi’s Southern Region. The Tanzanian study was conducted in northern Tanzania in the Kilimanjaro Region. The tertiary hospital, Kilimanjaro Christian Medical Centre (KCMC) is in the Moshi municipality which is the regional capital. It has endoscopy facilities. For the purpose of the study, other Kilimanjaro hospitals referred patients to KCMC. They were all within 70 km of KCMC: Hai and Siha District Hospitals, Huruma Hospital, Kibosho Hospital and Machame Lutheran Hospital. However, none of these other hospitals has endoscopy services, but Huruma has barium swallow capability. As some EC patients were too ill to travel to KCMC, interviewers travelled, at least monthly, to these outlying hospitals to conduct interviews.

### Eligibility criteria

For both countries, cases were those ≥18 years of age with a new ESCC diagnosis. In Malawi, all cases had an endoscopy pinch biopsy for histological examination and results that ruled out ESCC were excluded (e.g., adenocarcinoma). In Tanzania, histological confirmation was obtained for the majority of cases, but additional cases were included on the basis of imaging (barium swallow) or clinical criteria (difficulty swallowing solids/liquids and substantial weight loss). The inclusion of non-histologically confirmed cases can be justified because previous studies found that 86% of endoscopically diagnosed EC patients had obstruction, i.e., solid/liquid dysphagia symptoms are very specific [22]. Furthermore, studies from the Eastern African EC corridor indicate that the ESCC subtype comprised 90% or greater of EC [23, 24].

Recruitment of controls was carried out so as to ensure age and sex frequency matching with at least one control for every case. In both countries, adults aged 18 years of age or older who were inpatients, out-patients or visitors at the same hospitals as for case recruitment were approached and were invited to participate. Controls did not have cancer or digestive disease diagnosis.

### Questionnaire and exposure assessment

In both countries, a face-to-face questionnaire was administered by trained research assistants. Data regarding sociodemographic details, lifestyle and hot beverage/food consumption were collected in real time using a bespoke ESCCAPE study mobile phone application programmed by Mobenzi.

Concerning hot beverage/food thermal exposure metrics, participants from both countries were asked whether they ever consumed (in cases, before they were ill) each of the following hot beverages/food: tea, coffee and porridge (separately). If they answered yes to the question, they were then also asked a series of questions about their habits for that particular drink/food: the frequency of consumption, the perceived consumption temperature (‘warm’, ‘hot’, ‘very hot’ or ‘extremely hot’), how long they waited before drinking/consuming (>5, 2 to 5 and <2 minutes), the consumption speed (‘slow’, ‘normal’, ‘fast’ and ‘extremely fast’) and the average number of times their mouth/tongue was burnt per month. For tea and coffee, whether and when milk was added was also collected.

### Statistical analysis

Hot beverage drinking habits in controls were first examined, i.e., the closest available representation of the study’s source population. Chi-squared or Fisher’s exact tests were used, where appropriate, to determine if the beverage/food consumption temperature, dichotomised into very/extremely hot vs warm/hot, varied by known ESCC risk factors which could confound the association between thermal injury and ESCC. We then used logistic regression models to estimate the odds ratios (ORs) and their 95% confidence intervals (CI) of the association between thermal injury markers and risk of ESCC. All models were minimally adjusted for the design factors sex using a binary indicator and indicators for 5-year age categories as is advised by Breslow and Day [25]. Thereafter models were further adjusted for known risk factors for ESCC, including binary indicators for ever alcohol consumption and ever tobacco use. Finer adjustment for the intensity of alcohol or tobacco consumption was not deemed necessary as thermal exposures were not strongly correlated with any ESCC risk factors. Country-specific results were first examined and because they did not differ substantially, a combined model further adjusted for the country was also fitted.

Thermal injury metrics were fitted first as categorical variables, with the category corresponding to the lowest expected thermal injury as the reference, unless the number of subjects in this group was very low (<10 cases or controls in either country). These metrics were also fitted as a linear term, assigning scores as follows. For temperature: warm = 0, hot = 1, very hot = 2, extremely hot = 3; speed: slow = 0, normal = 1, fast = 2, extremely fast = 3; and waiting time in minutes (min): >5 min=1, 2 to 5 min = 2, <2 min = 3. We first analysed the above metrics separately for tea, coffee and porridge consumption. Thereafter, a participant was categorised under the highest temperature at which they consumed tea, coffee or porridge. A similar approach was applied for waiting time and speed, i.e., using the highest thermal exposure category across tea, coffee and porridge. For the number of mouth/tongue burns per month, the total burns across the three food items was calculated and then categories were assigned the following scores: less than two burns = 0, two burns = 1, three to five burns = 2 and six or more burns = 3. Finally, we generated a thermal exposure composite index, which was the sum of the temperature + speed + waiting time + mouth burning frequency scores for the three food items just specified. Individuals’ thermal exposure composite scores ranged from 1 to 12. We also investigated the possible interaction of hot beverage/food consumption with sex, age, alcohol and tobacco use using likelihood ratio tests for an interaction term between a unit increase in the thermal exposure index and each potential effect modifier. Finally, we conducted sensitivity analyses of the modelling approach, by comparing the OR obtained from a pooled analysis of data from the two countries (as described above) to that from a random-effects meta-analytic approach of the country-specific ORs. For statistical analysis, Stata version 17BE (StataCorp LP College Station, TX) was used.

## Results

### Participant and exposure characteristics

Recruitment occurred between November 2015 and December 2019 in Tanzania and between June 2017 and May 2020 in Malawi. During this time, in Tanzania 345 cases were approached of whom 322 (93%) agreed to participate. Due to a having non-ESCC histology, twelve consented cases were excluded, leaving 310 cases. The majority of these cases 244 (79%) were recruited at KCMC. In Malawi, 569 cases were approached and 553 (97%) agreed, of which 539 (97%) had histology which did not rule out ESCC. Control participation percentages were over 99% in Tanzania (313/314) and 94% (593/629) in Malawi. Among participating controls, in Tanzania most (76%) controls were hospital visitors, whilst 17% were hospital out-patients and 7% inpatients, whereas in Malawi, the corresponding percentages were 52, 12 and 36% respectively. Thus in total, the present analyses are based on 849 cases and 906 controls (Table 1). The mean age at diagnosis in Malawi was 57 years and 64 years in Tanzania. The male:female case ratio in Malawi was 1.4, and in Tanzania 3.2.

**Table 1:** Demographic and lifestyle characteristics of 849 cases and 906 controls: ESCCAPE Tanzania and Malawi esophageal squamous cell carcinoma case–control study.

		Malawi		Tanzania	
Characteristics		Cases, *n* (%)	Controls, *n* (%)	Cases, *n* (%)	Controls, *n* (%)
*N* (column %)		539	593	310	313
Sex	Male	311 (58)	335 (56)	237 (77)	237 (76)
	Female	228 (42)	258 (44)	73 (24)	76 (24)
Age (years) at diagnosis/interview	Mean (SD)	57 (±14)	56 (±15)	64 (±14)	62 (±14)
Religion^a^	Christian	445 (83)	526 (89)	187 (88)	201 (86)
	Muslim	59 (11)	50 (8)	24 (11)	33 (14)
	Other	34 (6)	17 (3)	2 (<1)	1 (<1)
Formal education	None	103 (19)	96 (16)	63 (20)	21 (7)
	Primary	310 (58)	311 (52)	207 (67)	231 (74)
	Secondary or higher	106 (20)	153 (26)	32 (10)	42 (13)
	Other	20 (4)	33 (6)	8 (3)	15 (5)
Marital status	Married	357 (66)	416 (70)	231 (75)	286 (91)
	Unmarried	182 (34)	177 (30)	79 (26)	27 (9)
Occupation	Farming	234 (43)	211 (36)	269 (87)	251 (80)
	Non-farming	305 (57)	382 (64)	41 (13)	62 (20)
Drank alcohol regularly^b^	No	281 (52)	341 (58)	75 (24)	171 (55)
	Yes	258 (48)	252 (43)	235 (76)	142 (45)
Ever smoked tobacco	No	316 (59)	452 (76)	129 (42)	259 (83)
	Yes	223 (41)	140 (24)	181 (58)	54 (17)

^a^This question was not initially asked in Tanzania and thus was systematically missing in that country for 78 controls and 97 cases. Percentages are among non-missing values.

^b^One drink per week for at least 6 months.

### Hot beverage and porridge consumption habits in controls

**Fig. 1:**
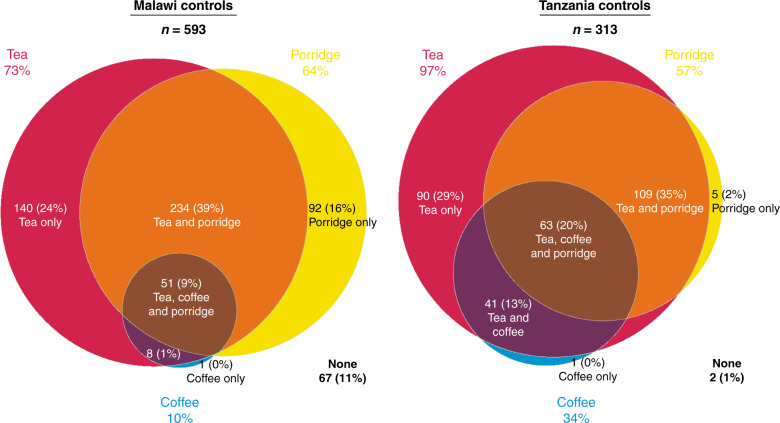
Distribution of hot beverage and porridge consumption in controls. Venn diagram of hot tea, coffee and porridge consumption among Malawi and Tanzanian controls.

Everyone except two controls (1%) consumed hot tea, coffee and/or porridge in Tanzania, whereas in Malawi, 67 (11%) controls did not consume any of these hot foods (Fig. 1)—proportionally, this Malawian group who did not consume any hot foods tended to be male (42/67 = 63%) compared to 56% (293/526) of hot food consumers who were men. Among controls, in both countries tea was the most commonly consumed hot beverage: 73% (433/593) of controls consumed tea in Malawi and 97% (303/313) in Tanzania. In Malawi, 10% of controls drank hot coffee, whilst this percentage was 34% in Tanzania. Consumption of hot porridge among controls was more similar across countries: Malawi (64%) and Tanzania (57%). In Malawi, the median age at which porridge consumption started was 5 years, tea at 10 years and coffee at 23 years, whilst in Tanzania consumers commenced at younger ages: 1, 5 and 15 years, respectively. Median daily tea drinking volumes were high in Tanzania for both coffee and tea (500 mL), with limited seasonal variation, whereas in Malawi, these were lower at 250 mL in warm and 300 mL in cooler months. The median number of porridge servings per week was three and two for Malawi and Tanzania respectively (Supplementary Fig. S1). Of the participants (cases and controls) who drank both tea and coffee, 91% (314/345) consumed both beverages at the same temperature, thus combining the hottest of tea or coffee temperature in later analyses typically reflects the same temperature. In Malawi among controls who drank milk (in tea or otherwise), several types of milk were used: 38% (140/377) used fresh cow’s milk, 36% (137/377) used processed in cartons or plastic sachets, 26% used powdered milk (99/377) and <1% fresh goat’s milk. In Tanzania among the 277 controls who drank milk, fresh cow’s milk was always used. Of the controls in Malawi who drank milky tea, 52% (45/86) prepared it by boiling the milk together with water, whereas this was always the habit in Tanzania. Almost all Tanzanians consumed tea from a thermal flask, whereas Malawians used flasks and teapots on the fire or table.

Among tea drinkers, correlates of drinking extremely/very-hot tea (vs warm/hot) are shown in Supplementary Table S1. A higher prevalence of extremely/very-hot drinkers was found in Tanzania men who drank alcohol regularly, but not in Tanzanian women or in Malawian participants. In Tanzanian men, extremely hot/very-hot drinking was also more common among those who ever smoked tobacco (*P* < 0.001), but not in other groups.

### Associations with ESCC risk

**Fig. 2:**
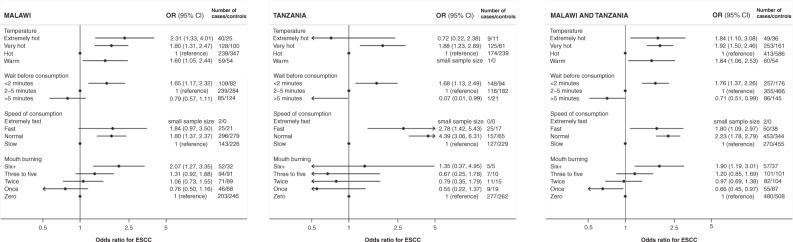
Associations of thermal exposure metrics with esophageal cancer risk. Odds ratios for ESCC, adjusted for age, sex, alcohol and tobacco, associated with categories of the highest temperature, faster drinking speed and shortest waiting time across habitual tea, coffee and porridge consumption, and for the total number of monthly mouth/burns across these food items.

In the models adjusted for tobacco smoking and alcohol consumption, all four thermal exposure measures were positively associated with ESCC risk. Consistent across countries, ‘very hot’ compared to ‘hot’ tea or coffee drinkers had almost a two-fold increase in ESCC risk: ORs were 1.89 (95% CI: 1.34, 2.67) and 1.93 (95% CI: 1.26, 2.96) in Malawi and Tanzania, respectively. These exposure groups were not rare: 24% of controls in both countries characterised themselves as ‘extremely/very hot’ drinkers of tea/coffee. Furthermore, those who consumed their tea or coffee within 2 min of pouring it into their cup/mug, compared to those who waited over 5 min, had an increased risk of ESCC: ORs were 1.56 (95% CI: 1.07, 2.27) and 1.26 (95% CI: 0.82, 1.93) in Malawi and Tanzania respectively (Table 2). Those who drank these beverages at a “normal” compared to a “slow” speed also had a raised ESCC risk with ORs of 1.97 (95% CI: 1.47, 2.64) and 2.24 (95% CI: 1.48, 3.39) in Malawi and Tanzania, respectively. Most participants in Tanzania (86%) reported no mouth/tongue burns in an average month, whereas in Malawi more than half of cases and controls reported at least one burn per month and in this country, an increasing number of burns was associated with raised ESCC risk: the OR for ≥6 burns per month vs. zero was 2.07 (95% CI: 1.27, 3.35), *P*-trend <0.01, Fig. 2). The positive associations with ESCC were generally similar in unadjusted and adjusted analyses, except in Tanzania where the unadjusted effects tended to be larger due to the aforementioned confounding. The associations with these metrics separately for tea (Supplementary Table S2) were similar to those of tea + coffee combined results.

**Table 2:** Odds ratios for esophageal cancer associated with thermal exposure metrics during consumption of hot beverages and porridge, in Malawi and Tanzania.

		Malawi		Tanzania		Malawi		Tanzania	
Characteristics		Cases, *n* (%)	Controls, *n* (%)	Cases, *n* (%)	Controls, *n* (%)	OR (95% CI)			
*N* (all participants)		539	593	310	313	Unadjusted	Adjusted*	Unadjusted	Adjusted*
Hot drinks (tea and/or coffee)									
* N* (drink hot tea and/or coffee drinkers)		399 (74)	434 (73)	298 (96)	306 (98)				
Temperature	Extremely hot	35 (9)	22 (5)	8 (3)	11 (4)	2.30 (1.29, 4.11)	2.29 (1.26, 4.14)	1.04 (0.41, 2.67)	0.61 (0.18, 2.04)
	Very hot	116 (29)	83 (19)	125 (42)	61 (20)	2.01 (1.44, 2.81)	1.89 (1.34, 2.67)	2.95 (2.04, 4.27)	1.93 (1.26, 2.96)
	Hot	204 (51)	290 (67)	164 (55)	234 (77)	1	1	1	1
	Warm	44 (11)	39 (9)	1 (0)	0 (0)	1.58 (0.99, 2.53)	1.59 (0.98, 2.60)	–	–
Number of minutes before drinking	<2	95 (24)	72 (17)	111 (37)	77 (25)	1.59 (1.11, 2.29)	1.56 (1.07, 2.27)	1.94 (1.34, 2.81)	1.26 (0.82, 1.93)
	2–5	198 (50)	230 (53)	139 (47)	187 (61)	1	1	1	1
	>5	81 (20)	106 (24)	4 (1)	28 (9)	0.88 (0.62, 1.25)	0.83 (0.58, 1.19)	0.19 (0.06, 0.55)	0.25 (0.08, 0.75)
	I do not know	25 (6)	26 (6)	8 (3)	1 (0)	1.08 (0.60, 1.93)	0.92 (0.51, 1.68)	11.5 (1.34, 99.1)	–
Speed of drinking	Extremely fast	2 (1)	0 (0)	0 (0)	0 (0)	–	–	–	–
	Fast	18 (5)	19 (4)	24 (8)	17 (6)	1.47 (0.75, 2.87)	1.46 (0.71, 3.01)	2.39 (1.22, 4.70)	1.28 (0.55, 2.98)
	Normal	220 (55)	177 (41)	130 (44)	58 (19)	1.87 (1.41, 2.49)	1.97 (1.47, 2.64)	3.63 (2.50, 5.28)	2.24 (1.48, 3.39)
	Slow	159 (40)	238 (55)	144(48)	231 (76)	1	1	1	1
Numbers of times burnt tongue or mouth per month	≥4	63 (16)	55 (13)	9 (3)	9 (3)	1.40 (0.92, 2,13)	1.36 (0.87, 2.11)	0.99 (0.39, 2.56)	1.15 (0.42, 3.11)
	3	42 (11)	39 (9)	2 (1)	2 (1)	1.32 (0.81, 2.14)	1.32 (0.80, 2.17)	0.92 (0.13, 6.76)	1.23 (0.19, 7.81)
	2	76 (19)	79 (18)	11 (4)	13 (4)	1.17 (0.80, 1.72)	1.30 (0.88, 1.92)	0.81 (0.36, 1.85)	0.81 (0.34, 1.93)
	1	49 (12)	67 (15)	7 (2)	19 (6)	0.87 (0.57, 1.33)	0.78 (0.51, 1.19)	0.35 (0.14, 0.85)	0.43 (0.17, 1.11)
	0	169 (42)	194 (45)	269 (90)	263 (86)	1	1	1	1
Porridge									
* N* (porridge consumers (%))		371 (69)	377 (64)	231 (75)	179 (57)				
Temperature	Extremely hot	8 (2)	3 (1)	1 (0)	1 (1)	2.85 (0.71, 11.5)	2.33 (0.53, 10.2)	–	–
	Very hot	62 (17)	40 (11)	4 (2)	4 (2)	1.67 (1.07, 2.60)	1.65 (1.06, 2.58)	–	0.60 (0.07, 5.27)
	Hot	195 (53)	211 (56)	225 (97)	173 (97)	1	1	1	1
	Warm	106 (29)	123 (33)	1 (0)	1 (1)	0.95 (0.68, 1.31)	0.98 (0.70, 1.37)	–	–
Number of minutes before eating	<2	41 (11)	23 (6)	114 (49)	56 (31)	1.86 (1.07, 3.22)	2.12 (1.22, 3.69)	2.83 (1.81, 4.43)	2.21 (1.36, 3.58)
	2–5	182 (49)	181 (48)	73 (32)	101 (57)	1	1	1	1
	>5	116 (31)	134 (36)	2 (1)	9 (5)	0.88 (0.63, 1.21)	0.90 (0.64, 1.27)	0.29 (0.06, 1.41)	0.43 (0.12, 1.54)
	I do not know	32 (9)	39 (10)	6 (3)	2 (1)	0.77 (0.46, 1.29)	0.72 (0.43, 1.22)	4.26 (0.87, 20.6)	2.14 (0.44, 10.4)
Speed of eating	Extremely fast	1 (0)	0 (0)	0 (0)	0 (0)	–	–	–	–
	Fast	10 (3)	2 (1)	15 (7)	6 (3)	5.57 (1.23, 24.4)	3.77 (0.89, 16.0)	4.40 (1.61, 12.1)	2.73 (0.78, 9.56)
	Normal	212 (57)	205 (54)	138 (60)	42 (24)	1.20 (0.90, 1.62)	1.28 (0.94, 1.72)	5.51 (3.53, 8.60)	3.83 (2.38, 6.15)
	Slow	148 (40)	170 (45)	78 (34)	131 (73)	1	1	1	1
Numbers of times burnt tongue or mouth per month	≥3	42 (11)	24 (6)	1 (0)	1 (1)	1.91 (1.13, 3.25)	1.89 (1.12, 3.18)	–	–
	2	30 (8)	30 (8)	2 (1)	5 (3)	1.04 (0.61, 1.76)	0.88 (0.52, 1.49)	0.29 (0.06, 1.49)	0.28 (0.08, 1.00)
	1	44 (12)	50 (13)	4 (2)	5 (3)	0.94 (0.61, 1.46)	0.95 (0.60, 1.51)	0.60 (0.16, 2.20)	0.76 (0.20, 2.88)
	0	255 (69)	273 (73)	224 (97)	168 (94)	1	1	1	1

*OR* odds ratio, *CI* confidence interval.

“Unadjusted” is adjusted for design factors 5-year age categories and sex. *Adjusted is further adjusted for smoking and alcohol consumption.

For porridge, in general, associations with each thermal injury metric were consistent with those for hot drinks where exposure contrasts were present. The ESCC OR for ‘very hot’ compared to ‘hot’ porridge consumers was 1.65 (95% CI: 1.06, 2.58) in Malawi. In Tanzania most people (97% of both cases and controls) consumed porridge at a ‘hot’ temperature, precluding comparison between categories. However, compared to those who waited more than 5 min, those who consumed porridge within 2 min had an increased risk of ESCC with ORs of 2.12 (95% CI: 1.22, 3.69) and 2.21 (95% CI: 1.36, 3.58) in Malawi and Tanzania respectively (Table 2). In total, 27% of porridge consumers reported burning their mouth at least once per month and those who burnt themselves three times or more per month had a 1.9-fold (1.1–3.2) increase in ESCC risk relative to porridge consumers who did not burn themselves.

In Table 2, we note that for most exposure metrics, categories with higher thermal injury were associated with increased ESCC risks in a monotonic increasing fashion for categories which had a sufficient number of participants. The only exception to this was a U-shaped association in Malawi, where ‘warm’ compared to ‘hot’ was expected to be protective but it had an OR 1.59 (95% CI: 0.98, 2.60) (Fig. 2) which was not significant at the 5% level. Upon further post hoc investigations, one possible explanation for this U-shape was interviewer differences in the temperature distributions which could not be adjusted for because some interviewers exclusively interviewed cases (Supplementary Table S3). When restricting the analysis to the two main interviewers who interviewed both cases and controls (176 cases excluded), ‘warm’ compared to ‘hot’ drinkers had an OR 0.87 (95% CI: 0.51, 1.46) which was in line with the anticipated trend (Supplementary Fig. S2). Similarly, the reduced OR associated with one compared to zero burns was completely attenuated to the null. These latter subset analyses are purely exploratory, especially because the 95% CIs in both instances include 1, thus variations in the ORs may purely have occurred due to random variation. Further, positive associations with ESCC risk were seen for all other thermal exposure metrics regardless of the interviewer combination.

**Fig. 3:**
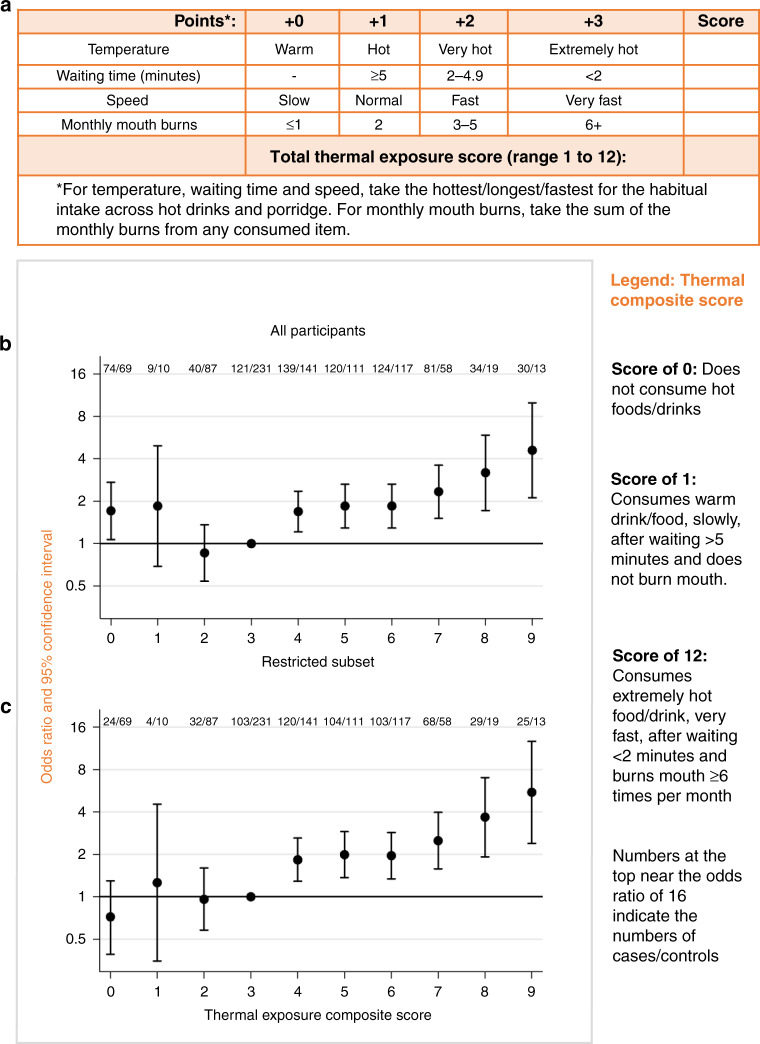
Esophageal squamous cell cancer risk and a 12-point thermal exposure index. **a** A score card to generate the 12-point thermal exposure composite score. A score of zero is for non-consumers of hot foods/beverages. **b** Odds ratios for ESCC risk associated with the thermal exposure composite score (Malawi and Tanzania combined). **c** Odds ratios for ESCC risk associated with the thermal exposure score based on all participants from Tanzania and participants in Malawi who were interviewed by the two researchers who interviewed both cases and controls. In **b** and **c**, scores of 9 through 12 were combined due to small numbers. The 30/13 cases/controls had scores of 9 (n = 19/10), 10 (n = 7/3), 11 (n = 3/0) and 12 (n = 1/0).

As seen in Table 2, the most common category often varied between countries but in general positive trends of higher ESCC risk with greater potential thermal injury were observed. Thus, to gain statistical power, trends were fitted per unit increase in the ordered categories (Table 3). Overall, the trends for all four thermal injury metrics were highly consistent between Malawi and Tanzania. Notably, the steepest gradient was observed for speed (OR 1.76 per category), then waiting time (OR 1.55) before drinking, then reported temperature (OR 1.33) and mouth burning (OR 1.19). For mouth/tongue burning, one burn per month was associated with a 7% (95% CI: 2–12) increase in ESCC risk. Similar positive associations across these metrics were explained by positive correlations among them. These were strongest for temperature and waiting time (Spearman’s correlation coefficient = 0.55, *P* < 0.001). Thus, for a more complete thermal exposure index composed of the sum of the temperature, speed, waiting time and mouth/tongue burning category scores. Figure 3 illustrates the make-up of this score (Fig. 3a), as well as its association with ESCC risk (Fig. 3b). The higher the composite score, the higher the ESCC risk. A unit increase in the score (in the range 1–12) had an ESCC OR of 1.22 (95% CI: 1.14, 1.30) in pooled analyses. This estimate was very similar to that obtained using a random-effects meta-analytic approach: OR 1.20 (1.12, 1.29) and there was no evidence of between-country heterogeneity (P = 0.29). In the pooled analysis, a thermal exposure composite score of 9 or more, compared to 3, was associated with a 4.6 (2.1, 10.0) fold increase in ESCC risk and strengthened further after restriction to the main interviewers in Malawi (Fig. 3c and Supplementary Table S5). The distribution of the composite score for controls is also depicted visually (Supplementary Fig. S3).

**Table 3:** Odds ratios for esophageal cancer associated with thermal exposure metrics among consumers of hot beverages and/or porridge in Malawi and Tanzania.

Metric	Number of cases/controls			OR (95% CI) for a unit increase in each thermal exposure metrics		
Per unit increase in category, unless otherwise state	Malawi	Tanzania	Both	Malawi	Tanzania	Malawi and Tanzania^a^
Higher drinking temperature	466/526	309/311	775/837	1.27 (1.06, 1.52)	1.38 (0.94, 2.01)	1.33 (1.13, 1.57)
Shorter waiting time before consumption^b^	433/490	265/297	698/787	1.43 (1.16, 1.76)	1.92 (1.35, 2.74)	1.55 (1.30, 1.84)
Faster drinking speed	466/526	309/311	775/837	1.64 (1.30, 2.08)	1.84 (1.30, 2.60)	1.76 (1.46, 2.11)
Mouth/tongue burning, per monthly burn	466/526	309/311	775/837	1.08 (1.03, 1.13)	0.99 (0.88, 1.11)	1.07 (1.02, 1.12)
Mouth/tongue burning, per category increase^c^	466/526	309/311	775/837	1.24 (1.09, 1.41)	0.96 (0.70, 1.32)	1.19 (1.06, 1.35)
				Per unit increase in a thermal exposure composite score^d^ (overall and in different study subsets)		
Overall	433/490	265/297	698/787	1.18 (1.10, 1.26)	1.29 (1.11, 1.50)	1.22 (1.14, 1.30)
By age						
<50 years	160/192	45/53	205/245	1.09 (0.99, 1.21)	1.72 (1.13, 2.62)	1.14 (1.03, 1.26)
50+ years	273/298	220/244	493/542	1.24 (1.13, 1.36)	1.20 (1.02, 1.42)	1.25 (1.16, 1.36)
* P*_interaction_				*P* = 0.35	*P* = 0.24	*P* = 0.57
By sex						
Male	243/272	206/229	449/501	1.17 (1.07, 1.28)	1.27 (1.06, 1.51)	1.22 (1.12, 1.32)
Female	190/218	59/68	249/286	1.19 (1.07, 1.34)	1.37 (1.04, 1.83)	1.23 (1.11, 1.36)
* P*_interaction_				*P* = 0.70	*P* = 0.58	*P* = 0.73
By control type						
Hospital visitors	433/257	265/228	698/485	1.13 (1.00, 1.26)	1.46 (1.23, 1.73)	1.27 (1.15, 1.40)
Hospital patients	433/233	265/69	698/302	1.42 (1.25, 1.61)	1.17 (0.92, 1.50	1.38 (1.24, 1.55)
By alcohol/tobacco use						
Never alcohol nor tobacco	200/272	49/158	249/430	1.12 (1.02, 1.23)	1.62 (1.25, 2.09)	1.18 (1.08, 1.29)
Ever alcohol and ever tobacco	142/80	141/46	283/126	1.33 (1.15, 1.55)	0.93 (0.72, 1.20)	1.20 (1.06, 1.36)
* P*_interaction_				*P* = 0.16	*P* < 0.001	*P* = 0.73

*OR* odds ratio, *CI* confidence interval.

All ORs are adjusted for age, sex, alcohol and tobacco.

^a^Adjusted for the country in addition.

^b^Trend across <2, 2–5 and 5+ minutes and omitting the “I do not know” category.

^c^Per category where zero/one burns = 0, two burns = 1, three to five burns = 2 and 6+ burns on average per month = 3.

^d^Sum of drinking temperature + waiting time + drinking speed + mouth burning categories, range 1–12.

The composite score was also used to investigate potential effect modifiers. There was no evidence of effect modification by sex (*P* = 0.73), or age (*P* = 0.57). For alcohol and tobacco as effect modifiers, the thermal exposure score had a stronger effect in alcohol and tobacco consumers in Malawi, but in abstainers in Tanzania (Table 3). Compared to their counterparts who drank black tea, milky tea drinkers had lower ESCC risks in Tanzania (OR 0.51 (95% CI: 0.33, 0.78)), whereas the opposite was seen in Malawi where milky tea drinkers had a 2.10-fold (95% CI: 1.50, 2.95) increased ESCC risk in Malawi. The higher risk for milky tea drinkers in Malawi held when restricting to the two main interviewers in that country (not shown). Further, in Malawi, drinking over 600 mL of tea per day compared to <200 mL was associated with increased ESCC risk: ORs were 4.16 (95% CI: 2.17, 8.00) and 2.73 (95% CI: 1.67, 4.46) in the hot and cooler seasons respectively (Supplementary Table S4).

## Discussion

### Principal findings

Primary prevention strategies for esophageal cancer are needed in Africa because the prognosis is extremely poor and this disease kills 26,000 people per year [1]. If thermal injury to the oesophagus is implicated, it may represent a modifiable factor with an easy to communicate prevention message. In the current analysis, we presented findings from Malawi and Tanzania regarding thermal exposures from the consumption of hot tea, coffee and porridge and ESCC risk. We found a consistent pattern across countries, ages and sexes, whereby ESCC risk was increased for higher consumption temperature, shorter time from serving to consumption, faster consumption speed and mouth/tongue burning from hot beverage/porridge consumption. Based on these four measures, we developed a composite thermal exposure index with a score ranging from 1 to 12. This index was strongly associated with ESCC risk, reaching an OR of 4.6 (2.1–10.0) for scores of ≥9 vs 3. The score thus provides an improved thermal exposure metric for future studies, for risk assessment and importantly provides evidence of a dose–response relationship. In relation to other characteristics of hot drink/porridge consumption, we did not observe consistent associations of ESCC risk with the age consumption commenced, but there was a suggestion of higher risk with larger volumes of consumption in Malawi. Consumers of black compared to milky tea had an increased ESCC risk in Tanzania, whereas the opposite was seen in Malawi. Effect modification by alcohol and tobacco were also not consistent between countries.

The results from the range of thermal exposure measures emphasise the importance of ascertaining both temperature and speed of consumption. These results are in line with observations that the intra-esophageal liquid temperature (IELT) [18] is affected as much by the sip temperature as by the sip volume because a greater speed of consumption must imply many small sips at a higher temperature or fewer larger sips. Interestingly, the 4-category exposure metric “speed of drinking” had the strongest association with ESCC risk and this is the metric where people can directly compare when they finish drinking compared to their peers, whereas one uncommonly directly experiences the temperature of a peer’s drink. The composite index combining four thermal exposure measures also benefits from reduced measurement error compared to any single measure, as can be seen from the narrower confidence intervals. Thus, in future studies, whilst real measurements of temperature and sip volume throughout the drinking experience are recommended if they are feasible and affordable, we recommend to include the range of exposure metrics included here for an improved thermal exposure assessment.

We previously hypothesised that consumption of milky tea (50% water, 50% full-fat milk) was an ESCC risk factor in Tanzania, based on the observation that milky tea was consumed hotter and cooled at a slower rate than black tea [19]. However, in this study in Tanzania, a lower ESCC risk was found among milky tea drinkers, which was partially but not fully due to milky tea drinkers drinking at lower thermal exposure levels. If the liquids had the same thermal energy, energy transfer to the oesophagus from milky tea might occur at a lower rate than from black tea [19]. On the other hand, the greater injury would be expected due to the fat content of milk, as seen in studies of skin burns in children [26]. Thus, the reason why milky tea drinkers in Tanzania, in the present study, had lower ESCC risk remains an open question. In contrast, in Malawi, those who consumed milky tea had an elevated ESCC risk compared to black tea drinkers—a finding which was not influenced by the interviewer effect. The different type of milk used in the two countries (fresh in Tanzania and processed/powdered in Malawi) might explain these differences, through a biological effect or a confounding bias due to a socially-patterned factor or residual confounding. In particular, in Malawi the habitual tea type had a strong social gradient whereby groups with a higher wealth status are more likely to drink milky tea. These observations require further study in other African studies.

### Comparison with other studies

Our findings are consistent with several previous observations from African and other settings. From ESCCAPE Kenya, where tea consumption is most prevalent, we previously reported similar findings for self-reported temperature [18]. A case–control study in Iran also found an association with self-reported temperature and waiting time [27] which were consistent with objective temperature measurements in a prospective design [16]. Mouth burning was relatively rare in Tanzania, in contrast to its habitual occurrence in some Asian settings [17]. In Malawi, mouth/tongue burning was more frequent, with 16% of controls reporting at least three burns per month and ESCC risk increased with more frequent mouth burning. This positive finding for burn frequency contrasts to the lack of evidence of an association in Kenyan [18] and previous Tanzanian studies [10]. However, the Kenyan study combined ‘do not know’ and ‘no’ burns and the Tanzanian study ascertained ever/never burns. Thus, the improved exposure assessment for the frequency of mouth burning in the present study may be an important factor to reveal the increased ESCC risk. Further, a reduced odds ratio for one vs no monthly burns was likely due to the interviewer imbalance as those who burnt once per month had higher thermal exposures of the other three metrics. Pain receptors are activated at high temperatures to protect the consumer from incurring more pain/thermal injury [28]. Finally, the increased risk with greater drinking volumes in Malawi are also consistent with those from Iran, where hot beverage volumes can also be very large [16].

### Strengths and limitations

The major strengths of the present investigation include the detailed exposure assessment for thermal exposures during hot beverage/food consumption, with several metrics, intensity-graded, assessed across a range of hot drinks and porridge. This allowed for investigations of dose–response associations, consistency across metrics and the generation of a more robust thermal exposure score with a greater exposure range. This exposure metric was found to be strongly associated with ESCC risk in a positive dose–response fashion. Nevertheless, a potential limitation inherent in the case–control study design is recall bias [29]. Cases with EC had more time to ponder the circumstances surrounding why they developed the disease. Their recall for factors like their consumption and perception of hot beverages/food may have changed due to developing the disease and suffering from dysphagia due to it. However, hot beverage drinking is not an established risk factor nor a highly suspected one often discussed in these communities. As positive associations were also observed in prospective studies and in studies of esophageal cancer precursors [30, 31], it is unlikely that our overall findings would have been entirely explained by such a recall bias. A further limitation is between-person variability in the perception of the thermal exposure metrics in the absence of absolute measurements, which would lead to exposure misclassification which may have attenuated results. Finally, there were differences between interviewers in the distribution of thermal metrics, as already seen in ESCCAPE Kenya [18]. This effect was compounded by the lack of a balanced number of cases and controls interviewed by each interviewer which we should have identified during the study implementation phase. Furthermore, the range of thermal injury circumstances ascertained may not have been complete. In addition to porridge (a cooked watery maize flour mixture), the staple crop maize is consumed as a hot cooked stiff dough-like food, known as *nsima*, *pap*, *sadza* and *ugali*, depending on country/region. This was not captured in the present study. Other generic strengths of the ESCCAPE case–control studies are the histologically confirmed diagnosis of most ESCC cases, reducing outcome misclassification. Additionally, whilst selection bias is often a problem in cancer case–control studies, especially in LMICs due to differences in sociodemographic factors and/or urban/rural residential location between cases and controls, bias would only be introduced if thermal exposures were associated with these factors. This is not a major concern for thermal exposure as in this and previous studies in East Africa, there are no suggestions that there is a social/residential gradient of hot beverage consumption temperatures/volumes.

### Biological plausibility and public health implications

Chronic thermal injury to the oesophagus is a plausible carcinogen due to several related observations. First, concerning other tissues, for the skin, severe thermal injuries leading to burn scars associated with Marjolin’s ulcer can lead to squamous cell carcinomas. In the oesophagus, acute discomfort after consumption of extremely hot/boiling beverages manifests as candy-cane oesophagus upon endoscopic examination, which is reversible but does illustrate localised inflammation of this tissue at high consumption temperatures. Whilst habitual consumption is typically at lower temperatures, this chronic habit might lead to repeated irritation of the esophageal mucosa or an altered esophageal microbiome. Inflammation can lead to the endogenous production of reactive nitrogen species including nitrosamines. Alternatively, a damaged mucosa may expose the epithelium to other carcinogens. Further, a higher prevalence of p53 mutations (G:C to A:T mutations at CpG sites) has been found in esophageal tumours in patients with greater thermal exposures [32].

Primary prevention strategies for ESCC in Africa are needed. To date, a clear role of alcohol and tobacco has been established in some African settings and especially in men, but for the substantial proportion of abstainers from these habits, other measures are needed. The present findings indicate that the thermal injury pathway may be a significant contributor to ESCC risk, as consumption of hot beverage/food is commonplace in adults, thus cancer control plans should consider advice to reduce this exposure [6]. Lowering the beverage/food consumption temperature, allowing beverages to cool, sipping slowly and ensuring not to burn oneself whilst consuming hot food and drink are advisable. Fortunately, these are cheap easy messages to convey. Future research is needed to evaluate whether individuals are willing to change their habits. Finally, a research gap remains as to how early in life thermal injuries might commence, especially in settings where children consume tea from very young ages and given the unusually large burden of young ESCC cases in East Africa.

## Supplementary information

Requested reproducibility checklist

Supplementary Material

## Data Availability

Data are available and can be accessed via collaboration with IARC (https://esccape.iarc.fr/en/Contact).
