# Supplementary material for: A very-hot food and beverage thermal exposure index and esophageal cancer risk in Malawi and Tanzania: findings from the ESCCAPE case–control studies
Source: Br J Cancer. 2022 Jun 29;127(6):1106–15. doi: 10.1038/s41416-022-01890-8 (PMC9470732; doi:10.1038/s41416-022-01890-8)
Supplement: Supplementary file 2 — Supplementary Material [file 41416_2022_1890_MOESM2_ESM.docx]

**Supplementary appendix**

**Hot beverage and food thermal exposure metrics and esophageal cancer risk in Malawi and Tanzania: Findings from the ESCCAPE case–control studies**

Gwinyai Masukume^1^, Blandina T Mmbaga^2^, Charles P Dzamalala^3^, Yohannie B Mlombe^3^, Peter Finch^3^, Gissela Nyakunga^2^, Alex Mremi^2^, Daniel RS Middleton^1^, Clement T Narh^1,4,^ Steady JD Chasimpha^5^, Behnoush Abedi-Ardekani^6^, Diana Menya^7^, Joachim Schüz^1^, Valerie McCormack^1^

**Contents**

**Supplementary Fig. S1.** A. Age (years) consumption begins B. Volumes (mL) consumed per day. C Number of servings per week………………………………………………….………..2

**Supplementary Fig. S2**. Odds ratios (OR) and 95% confidence intervals (CI) for the association of self-reported tea, coffee or porridge consumption (hottest of the three) characteristics with esophageal cancer risk in Malawi and combined with Tanzania. Restricted to two interviewers in Malawi who interviewed both cases and controls.…………………………………...………………………………………………..….3

**Supplementary Fig. S3.** The composite thermal exposure score in Malawi and Tanzania in controls. Zero (0) denotes those who do not consume tea, coffee or porridge. ………….……4

**Supplementary Table S1.** Prevalence of self-reported tea drinking at ‘extremely’ or ‘very hot’ temperatures (as opposed to ‘hot’ or ‘warm’) among tea drinkers in controls, overall and by potential confounding factors and other beverage characteristics…………………………….5

**Supplementary Table S2.** Exposure characteristics of cases and controls in tea drinkers and odds ratios for squamous esophageal cancer risk….……………………………………….…6

**Supplementary Table S3.** Number of cases and controls interviewed by each interviewer in Malawi…………………………………………….………………………………………..….7

**Supplementary Table S4.** Odds ratios for esophageal cancer associated with further tea drinking characteristics in Malawi and Tanzania………………………………………………8

**Supplementary Table S5.** Odds ratios for the composite thermal exposure score…....……………………………………………………………………………..……….9

**Supplementary Fig. S1.** A. Age (years) consumption begins. B. Volumes (mL) consumed per day. C. Number of servings per week. The median and interquartile ranges are reported below each food type.


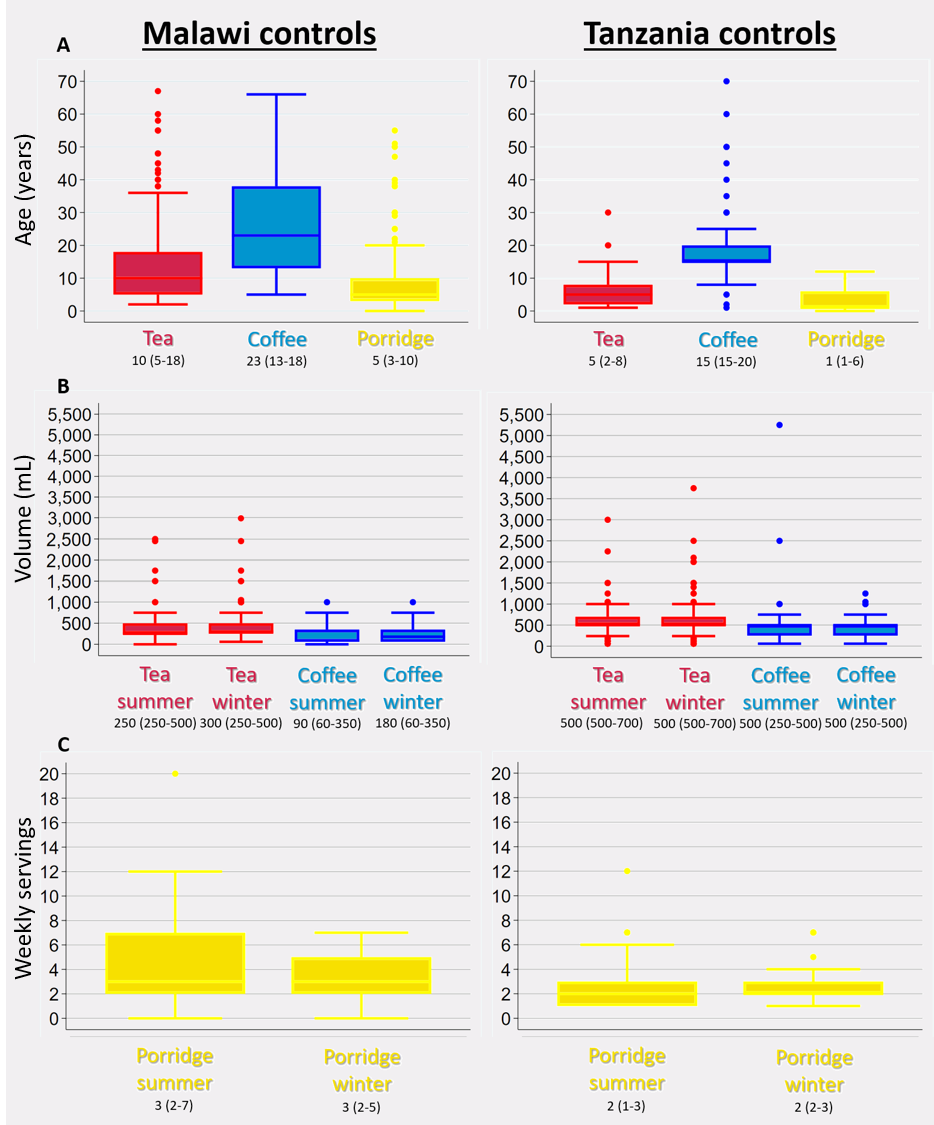


**Supplementary Fig. S2**. Odds ratios (OR) and 95% confidence intervals (CI) for the association of self-reported consumption characteristics of tea, coffee or porridge (hottest of the three) with esophageal cancer risk in Malawi and combined with Tanzania adjusted for tobacco, alcohol use and country when countries combined. Analyses for Malawi are restricted to two interviewers who interviewed both cases and controls.
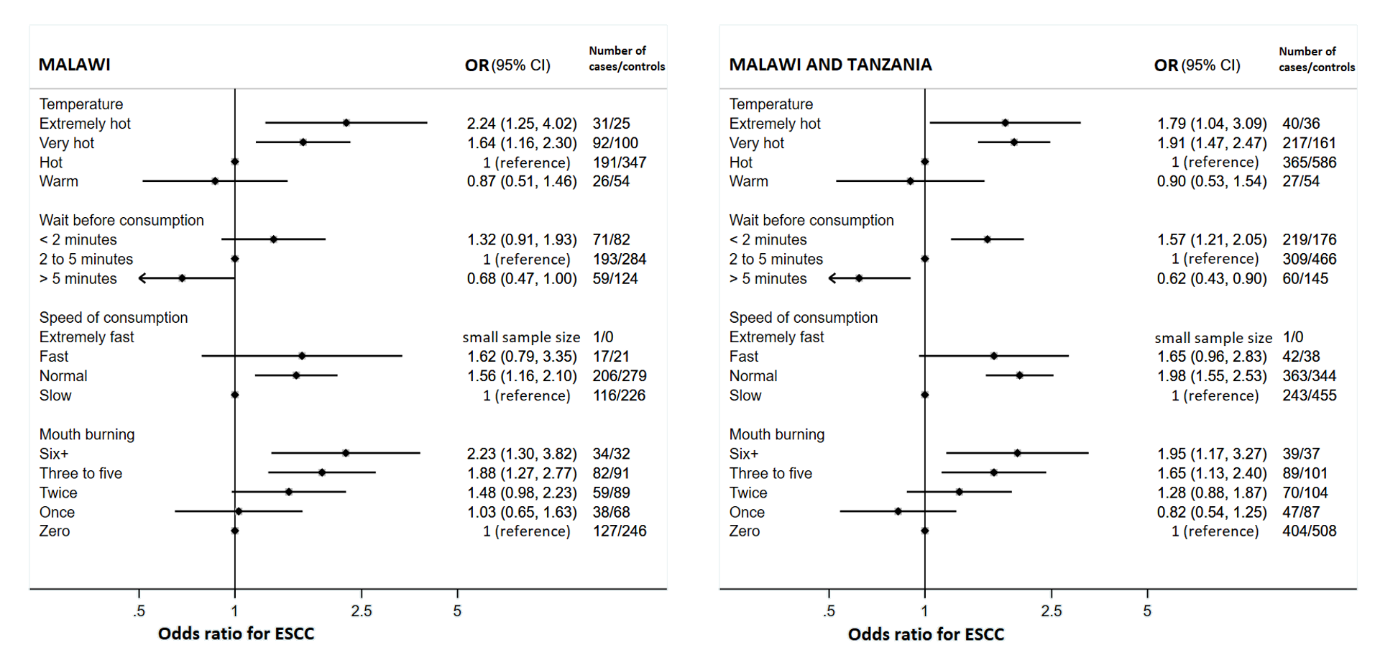


.

**Supplementary Fig. S3.** The composite thermal exposure score in Malawi and Tanzania in controls. A score of 0 denotes those who do not consume tea, coffee or porridge. Among consumers, the score ranges from 1 (consumes “warm” drinks “slowly” after “waiting over 5 minutes” and “never burns” mouth) to 12 (consumes “extremely hot” drinks “extremely fast” after “waiting under 2 minutes” and “ burns mouth” at least 6 times per month). No controls had scores of 11 and 12.

**Supplementary Table S1.** Prevalence of self-reported tea drinking at ‘extremely’ or ‘very hot’ temperatures (as opposed to ‘hot’ or ‘warm’) among tea drinkers in controls, overall and by potential confounding factors and other beverage characteristics.

|  |  | Malawi |  | Tanzania |  |
| --- | --- | --- | --- | --- | --- |
|  |  | Stratum-specific prevalence of drinking at ‘extremely’ or ‘very hot’ temperatures (as opposed to ‘hot’ or ‘warm’) among hot tea drinkers and p-value for difference between strata: N (row %) | | | |
| Overall |  | 104/433 (24%) | p-value^a^ | 68/303 (22%) | p-value^a^ |
| Sex | Male | 55/249 (22) | 0.27 | 56/232 (24) | 0.20 |
|  | Female | 49/184 (27) |  | 12/71 (17) |  |
| Age (years) | < 40 | 12/61 (20) | 0.06 | 2/19 (11) | 0.03 |
|  | 40 to < 60 | 59/202 (29) |  | 31/98 (32) |  |
|  | ≥ 60 | 33/170 (19) |  | 35/186 (19) |  |
| Type of tea | Milky tea | 15/86 (17) | 0.27 | 36/246 (15) | < 0.001 |
|  | Black tea (no milk) | 83/326 (23) |  | 28/51 (55) |  |
|  | Other | 6/21 (29) |  | 4/6 (67) |  |
| Type of milk | Fresh cow’s milk | 31/118 (26) | 0.27 | 49/266 (18) | <0.001 |
|  | Processed milk in cartons/sachets | 24/109 (22) |  | 0/0 (0) |  |
|  | Powdered milk | 27/90 (30) |  | 0/0 (0) |  |
|  | I do not drink milk | 22/116 (19) |  | 19/37 (51) |  |
| Tea cup size | Small 60 mL | 20/98 (20) | 0.17 | 14/21 (66) | < 0.001 |
|  | Medium 250 mL | 32/158 (20) |  | 22/193 (11) |  |
|  | Large mug 350 mL | 21/77 (27) |  | 1/27 (4) |  |
|  | Large cup 500 mL | 31/100 (31) |  | 0/1 (0) |  |
|  | I don't know | 0/0 (0) |  | 3/3 (100) |  |
|  | Not asked | - |  | 28/58 (48) |  |
| How tea is stored before it is poured into a cup | Tea pot on table | 15/74 (20) | 0.36 | 2/4 (50) | 0.002 |
|  | Tea pot on a fire | 43/199 (22) |  | 2/2 (100) |  |
|  | In a thermal flask | 45/156 (29) |  | 61/293 (21) |  |
|  | Other | 1/4 (25) |  | 3/4 (75) |  |
| Age (years) when one started drinking tea | < 5 | 15/36 (42) | 0.009 | 41/92 (45) | < 0.001 |
|  | 5 to < 10 | 30/165 (18) |  | 14/178 (8) |  |
|  | ≥ 10 | 59/232 (25) |  | 13/33 (39) |  |
| Education | None | 7/57 (12) | 0.17 | 8/20 (40) | 0.30 |
|  | Primary | 57/220 (26) |  | 48/224 (21) |  |
|  | Secondary | 32/126 (25) |  | 8/40 (20) |  |
|  | Other | 8/30 (27) |  | 4/19 (21) |  |
| Marital status | Married | 75/312 (24) | 0.99 | 59/277 (21) | 0.12 |
|  | Unmarried | 29/121 (24) |  | 9/26 (35) |  |
| Occupation | Farming | 30/134 (22) | 0.43 | 57/244 (23) | 0.44 |
|  | Non farming | 74/299 (25) |  | 11/59 (19) |  |
| Drank alcohol regularly^b^, among men | No | 22/100 (22) | 0.98 | 14/122 (11) | < 0.001 |
|  | Yes | 33/149 (23) |  | 42/110 (38) |  |
| Drank alcohol regularly^b^, among women | No | 42/160 (26) | 0.76 | 7/45 (16) | 0.75 |
|  | Yes | 7/24 (29) |  | 5/26 (19) |  |
| Ever smoked tobacco, men | No | 34/172 (20) | 0.19 | 28/180 (16) | < 0.001 |
|  | Yes | 21/77 (27) |  | 28/52 (54) |  |
| Ever smoked tobacco, women | No | 48/177 (27) | 1.00 | 12/71 (17) | - |
|  | Yes | 1/6 (17) |  | 0/0 (0) |  |

^a^ Pearson’s Chi-squared or Fisher’s exact test; ^b^ one drink per week for 6 months

**Supplementary Table S2.** Associations of thermal exposures during tea drinking with risk of ESCC in Tanzania and Malawi.

|  |  | Malawi | | Tanzania | | Malawi | | Tanzania | |
| --- | --- | --- | --- | --- | --- | --- | --- | --- | --- |
| Characteristics |  | **Cases**  **n (%)** | **Controls**  **n (%)** | **Cases**  **n (%)** | **Controls**  **n (%)** | **OR (95% CI)** | | | |
| N |  | 539 | 593 | 310 | 313 | Minimally adjusted* | Adjusted** | Minimally adjusted* | Adjusted** |
| N (%) who consume hot tea | | 397 (74) | 433 (73) | 289 (93) | 303 (97) |  |  |  |  |
| Tea temperature | Extremely hot | 35 (9) | 22 (5) | 7 (2) | 11 (4) | 2.30 (1.29, 4.10) | 2.29 (1.27, 4.13) | 0.90 (0.34, 2.38) | 0.55 (0.15, 1.95) |
|  | Very hot | 114 (29) | 82 (19) | 113 (39) | 57 (19) | 2.00 (1.43, 2.80) | 1.88 (1.33, 2.66) | 2.82 (1.93, 4.12) | 1.91 (1.23, 2.97) |
|  | Hot | 204 (51) | 290 (67) | 168 (58) | 235 (78) | 1 | 1 | 1 | 1 |
|  | Warm | 44 (11) | 39 (9) | 1 (0) | 0 (0) | 1.58 (0.99, 2.53) | 1.59 (0.98, 2.60) | empty | empty |
| Number of minutes before drinking tea | Less than 2 | 94 (24) | 69 (16) | 102 (35) | 72 (24) | 1.69 (1.17, 2.43) | 1.64 (1.13, 2.40) | 1.95 (1.34, 2.85) | 1.27 (0.82, 1.97) |
|  | 2 to 5 | 195 (49) | 231 (53) | 137 (47) | 187 (62) | 1 | 1 | 1 | 1 |
|  | More than 5 | 83 (21) | 106 (25) | 6 (2) | 31 (10) | 0.92 (0.65, 1.31) | 0.87 (0.61, 1.25) | 0.26 (0.11, 0.66) | 0.35 (0.14, 0.89) |
|  | I don't know | 25 (6) | 27 (6) | 8 (3) | 1 (0) | 1.06 (0.59, 1.89) | 0.92 (0.51, 1.66) | 11.4 (1.33, 96.9) | 5.64 (0.60, 52.6) |
| Speed of drinking tea | Extremely fast | 2 (1) | 0 (0) | 0 (0) | 0 (0) | empty | empty | empty | empty |
|  | Fast | 18 (5) | 19 (4) | 24 (8) | 17 (6) | 1.92 (1.04, 3.54) | 1.84 (0.97, 3.50) | 2.78 (1.42, 5.43) | 1.50 (0.66; 3.46) |
|  | Normal | 214 (54) | 171 (40) | 126 (44) | 55 (18) | 1.70 (1.30; 2.23) | 1.80 (1.37; 2.37) | 4.39 (3.06, 6.31) | 2.79 (1.87; 4.19) |
|  | Slow | 163 (41) | 243 (56) | 139 (48) | 231 (76) | 1 | 1 | 1 | 1 |
| Numbers of times tongue or mouth burnt per month from tea | Thrice or more | 97 (24) | 89 (21) | 4 (1) | 8 (3) | 1.33 (0.93, 1.91) | 1.31 (0.90, 1.98) | 0.48 (0.14, 1.61) | 0.82 (0.26, 2.64) |
|  | Twice | 80 (20) | 79 (18) | 15 (5) | 13 (4) | 1.23 (0.84, 1.80) | 1.34 (0.91, 1.98) | 1.14 (0.53, 2.45) | 1.02 (0.47, 2.24) |
|  | Once | 51 (13) | 71 (16) | 7 (2) | 18 (6) | 0.85 (0.56, 1.29) | 0.78 (0.51, 1.19) | 0.38 (0.15, 0.93) | 0.41 (0.15, 1.13) |
|  | Zero | 169 (43) | 194 (45) | 263 (91) | 264 (87) | 1 | 1 | 1 | 1 |

*Adjusted for design factors: age categories and sex. ** Adjusted for age, sex, tobacco smoking and alcohol consumption

**Supplementary Table S3.** Number of cases and controls interviewed by each interviewer in Malawi

| **Interviewer** | **Number of cases** | **Number of controls** |
| --- | --- | --- |
| Interviewer 1 | 112 | 181 |
| Interviewer 2 | 251 | 412 |
| Interviewer 3 | 76 | 0 |
| Interviewer 4 | 23 | 0 |
| Interviewer 5 | 22 | 0 |
| Interviewer 6 | 35 | 0 |
| Other interviewers | 20 | 0 |

**Supplementary Table S4**. Odds ratios for esophageal cancer associated with further tea drinking characteristics in Malawi and Tanzania.

|  |  | Malawi | | Tanzania | | Malawi | | Tanzania | |  |
| --- | --- | --- | --- | --- | --- | --- | --- | --- | --- | --- |
| Characteristics |  | **Cases**  **n (%)** | **Controls**  **n (%)** | **Cases**  **n (%)** | **Controls**  **n (%)** | **OR (95% CI)** | | | |  |
| N |  | 397 | 433 | 289 | 303 | Adjusted for alcohol and tobacco | Additionally adjusted for the thermal exposure score | Adjusted for alcohol and tobacco | Additionally adjusted for the thermal exposure score | |
| Age (years) started drinking tea | < 5 | 23 (6) | 36 (8) | 132 (46) | 92 (30) | 0.79 (0.45, 1.41) | 0.69 (0.37, 1.28) | 1.33 (0.88, 2.02) | 1.09 (0.69, 1.72) | |
|  | 5 to 9 | 133 (34) | 165 (38) | 117 (41) | 178 (59) | 1 | 1 | 1 | 1 | |
|  | 10 to 14 | 71 (18) | 80 (19) | 33 (11) | 26 (9) | 1.09 (0.73, 1.63) | 1.12 (0.74, 1.69) | 1.34 (0.72, 2.51) | 1.03 (0.50, 2.15) | |
|  | ≥ 15 | 170 (43) | 152 (35) | 7 (2) | 7 (2) | 1.38 (0.99, 1.94) | 1.37 (0.97, 1.94) | 0.88 (0.26, 2.98) | 0.59 (0.12, 2.93) | |
| Type of tea | Black tea (no milk) | 262 (66) | 326 (75) | 113 (39) | 51 (17) | 1 | 1 | 1 | 1 | |
|  | Milky tea | 128 (32) | 86 (20) | 169 (59) | 246 (81) | 2.10 (1.50, 2.95) | 2.24 (1.58, 3,16) | 0.51 (0.33, 0.78) | 0.57 (0.35, 0.92) | |
|  | Other | 7 (2) | 21 (5) | 7 (2) | 6 (2) | 0.49 (0.20, 1.24) | 0.54 (0.22, 1.32) | 1.34 (0.40, 4.53) | 1.32 (0.41, 4.23) | |
| Volume of hot tea per day during hot season | < 200 mL | 69 (17) | 103 (24) | 59 (20) | 20 (7) | 1 | 1 | 1 | 1 | |
|  | 200 to < 400 mL | 203 (51) | 201 (46) | 19 (7) | 31 (10) | 1.43 (0.99, 2.08) | 1.38 (0.93, 2.05) | 0.25 (0.10, 0.59) | 0.28 (0.12, 0.69) | |
|  | 400 to < 600 mL | 76 (19) | 111 (26) | 54 (19) | 128 (42) | 0.98 (0.63, 1.53) | 0.83 (0.52, 1.33) | 0.19 (0.09, 0.40) | 0.22 (0.10, 0.47) | |
|  | ≥ 600 mL | 48 (12) | 18 (4) | 71 (25) | 63 (21) | 4.16 (2.17, 8.00) | 3.64 (1.84, 7.21) | 0.59 (0.28, 1.27) | 0.65 (0.30, 1.40) | |
| Volume of hot tea during winter/cooler season per day | < 200 mL | 65 (16) | 95 (22) | 55 (19) | 20 (7) | 1 | 1 | 1 | 1 | |
|  | 200 to < 400 mL | 156 (39) | 184 (43) | 20 (7) | 13 (4) | 1.15 (0.77, 1.70) | 1.14 (0.75, 1.72) | 0.62 (0.24, 1.63) | 0.64 (0.24, 1.68) | |
|  | 400 to < 600 mL | 83 (21) | 107 (25) | 43 (15) | 134 (44) | 1.15 (0.74, 1.79) | 1.08 (0.67, 1.73) | 0.15 (0.07, 0.33) | 0.18 (0.08, 0.40) | |
|  | ≥ 600 mL | 92 (23) | 47 (11) | 85 (29) | 75 (25) | 2.73 (1.67, 4.46) | 2.38 (1.41, 4.00) | 0.62 (0.29, 1.32) | 0.67 (0.32, 1.44) | |

**Supplementary Table S5.** Odds ratios for esophageal squamous cell carcinoma associated with a composite thermal exposure score (range 1 to 12), which is the sum of (temperature score (1 to 4) + speed (1 to 4) + waiting time (1 to 3) + mouth burning (1 to 4) -3), adjusted for age, sex, tobacco, alcohol use and country. ESCCAPE case-control studies in Malawi and Tanzania.

|  | All interviewers (full dataset)  Malawi and Tanzania | | Malawi and Tanzania  with analyses in Malawi restricted to interviewers who interviewed both cases and controls | |
| --- | --- | --- | --- | --- |
| Score | Cases / Controls | OR (95% CI) | Cases / Controls | OR (95% CI) |
| **Non-hot beverage/porridge consumers** | 74 / 69 | 1.71 (1.07, 2.73) | 24 / 69 | 0.72 (0.39, 1.32) |
| **1** | 9 / 10 | 1.85 (0.69, 4.96) | 4 / 10 | 1.26 (0.35, 4.55) |
| **2** | 40 / 87 | 0.86 (0.54, 1.36) | 32 / 87 | 0.96 (0.58, 1.60) |
| **3 (Ref)** | 121 / 231 | 1 | 103 / 231 | 1 |
| **4** | 139 / 141 | 1.69 (1.21, 2.36) | 120 / 141 | 1.83 (1.29, 2.61) |
| **5** | 120 / 111 | 1.85 (1.29, 2.64) | 104 / 111 | 1.99 (1.37, 2.90) |
| **6** | 124 / 117 | 1.85 (1.29, 2.65) | 103 / 117 | 1.96 (1.34, 2.86) |
| **7** | 81 / 58 | 2.34 (1.51, 3.60) | 68 / 58 | 2.50 (1.58, 3.98) |
| **8** | 34 / 19 | 3.19 (1.72, 5.90) | 29 / 19 | 3.68 (1.92, 7.02) |
| **9-12*** | 30 / 13 | 4.60 (2.12, 10.0) | 25 / 13 | 5.52 (2.39, 12.7) |

OR - Odds Ratio, CI – confidence interval
